# Integrated Production, Genomic Safety, Quality, and Stability Characterization of a Human-Derived *Limosilactobacillus reuteri* ATA-LTC-Lr050600 Strain for Probiotic Applications

**DOI:** 10.3390/microorganisms14102143

**Published:** 2026-09-23

**Authors:** Özlem Türkoğlu, İsmail Aslan, Ertugrul Osman Bursalioglu, Kübra Can Kurt, Şükran Özdatlı Kurtuluş, Ayşe Nilhan Atsü, Hediye Eker, Mehmet Onur Gökalp, Hilmi Bakır, Ahmet Arif Kurt

**Affiliations:** 1 Clinic of Radiology, Sancaktepe Şehit Prof. Dr. İlhan Varank Training and Research Hospital, Istanbul 34785, Türkiye; 2 Department of Pharmaceutical Technology, Hamidiye Faculty of Pharmacy, University of Health Sciences, Istanbul 34668, Türkiye; ismail.aslan@sbu.edu.tr; 3 ATA BIO Technology Pharmaceutical Industry and Trade Ltd., Ahmet Yesevi, Kerem St. No. 9, Pendik, Istanbul 34903, Türkiye; 4 Department of Medical Services and Techniques, Vocational School of Health Services, Sinop University, Sinop 57000, Türkiye; ebursalioglu@sinop.edu.tr; 5 Department of Basic Medical Sciences, Hamidiye Faculty of Dentistry, University of Health Sciences, Istanbul 34668, Türkiye; kubra.cankurt@sbu.edu.tr; 6 Department of Pharmaceutical Toxicology, Hamidiye Faculty of Pharmacy, University of Health Sciences, Istanbul 34668, Türkiye; sukranozdatli.kurtulus@sbu.edu.tr; 7 Vocational School, Istanbul Kent University, İstanbul 34406, Türkiye; aysenilhan.atsu@kent.edu.tr; 8 Department of Dermatology, Başakşehir Çam and Sakura City Hospital, Istanbul 34480, Türkiye; hediye.eker@sbu.edu.tr (H.E.); onur.gokalp@sbu.edu.tr (M.O.G.); 9 Department of Pharmacy Services, Vocational School of Health Services, Üsküdar University, Istanbul 34662, Türkiye; hilmi.bakir@uskudar.edu.tr; 10 Department of Pharmaceutical Technology, Faculty of Pharmacy, Suleyman Demirel University, Isparta 32000, Türkiye

**Keywords:** probiotic, whole-genome sequencing, microbiological quality, lyophilization, real-time stability, OECD toxicity study

## Abstract

Probiotic quality and safety require strain-specific characterization supported by controlled production and comprehensive evaluation. This study aimed to integrate controlled fermentation and biomass production, downstream processing and lyophilization, microbiological quality control, batch reproducibility, whole-genome characterization, in vitro functional and safety-related assessment, real-time stability monitoring, and OECD-compliant in vivo safety evaluation of the human-derived *Limosilactobacillus reuteri* strain ATA-LTC-Lr050600. The strain was produced under controlled fermentation conditions, followed by harvesting, protective formulation, and lyophilization. Product quality was assessed by standardized microbiological testing, whole-genome sequencing, and in vitro assays evaluating acid and bile tolerance, adhesion to a fibroblast cell model, hemolytic activity, and cytotoxicity. Real-time stability was monitored during storage, and safety was further evaluated using OECD-compliant acute and repeated-dose oral toxicity studies. The production process generated a lyophilized preparation with consistent microbiological quality and reproducible manufacturing characteristics. Whole-genome sequencing confirmed strain identity and did not detect acquired antimicrobial resistance determinants. The strain tolerated simulated gastrointestinal conditions, adhered to the evaluated fibroblast cell model, and showed no detectable hemolytic activity or cytotoxicity under the experimental conditions. Product viability and microbiological quality were maintained throughout the monitored storage period. In the in vivo studies, no treatment-related adverse effects were identified, and the repeated-dose study established a no-observed-adverse-effect level of 1000 mg/kg/day under the conditions examined. Collectively, these integrated findings provide a basis for further strain-specific evaluation and support the continued development of *L. reuteri* ATA-LTC-Lr050600 as a well-characterized probiotic candidate under the conditions examined.

## 1. Introduction

Probiotics are defined as live microorganisms that, when administered in adequate amounts, confer a health benefit on the host [1,2,3]. They are increasingly incorporated into functional foods and other health-related applications because of their potential to support host well-being [2,4]. However, probiotic characteristics cannot be generalized at the species level because functional efficacy, technological performance, host interactions, and safety profiles may vary substantially among individual strains [5]. Internationally accepted recommendations therefore emphasize that candidate probiotic strains should undergo comprehensive strain-level characterization, including accurate taxonomic identification, documentation of functional properties, and safety assessment for the intended use [1]. The recent taxonomic reclassification of the former genus Lactobacillus has further underscored the importance of genome-based identification and standardized nomenclature in ensuring strain traceability and scientific reproducibility [6]. In this context, several species, including *Limosilactobacillus reuteri* (formerly Lactobacillus reuteri), have attracted considerable interest because of their host-associated distribution and strain-dependent biological characteristics [6,7].

*L. reuteri* is a heterofermentative lactic acid bacterium that naturally colonizes the gastrointestinal tract of humans and a wide range of vertebrate hosts [5]. Its distribution reflects a high degree of host adaptation and ecological specialization. Owing to its diverse metabolic capabilities, this species has become one of the most extensively studied probiotic microorganisms and has been associated with processes that contribute to intestinal homeostasis, including modulation of host immune responses, maintenance of epithelial barrier integrity, and adaptation to the gastrointestinal environment [8,9]. Selected strains can also produce biologically active metabolites, most notably reuterin, which contributes to the antimicrobial potential and technological relevance of the species in food and probiotic applications [10,11]. Nevertheless, substantial genomic and phenotypic diversity exists among *L. reuteri* strains; their functional, technological, and safety characteristics should therefore be established individually rather than inferred at the species level [9]. Realizing the biological potential of *L. reuteri* in probiotic applications thus depends not only on strain selection but also on controlled and reproducible manufacturing processes that preserve its functional and technological properties [12,13].

The successful development of a probiotic strain extends beyond the demonstration of biological activity and requires a manufacturing process capable of consistently delivering a safe, viable, and functionally stable product [14,15]. Commercial probiotic production involves an integrated workflow that begins with the establishment and maintenance of a well-characterized microbial cell bank and proceeds through controlled seed preparation, fermentation, biomass production, cell harvesting, downstream processing, formulation, and long-term storage [12]. After fermentation, concentrated cells are typically recovered by centrifugation and stabilized by adding suitable protective agents before freezing and lyophilization. The dried biomass is subsequently blended, packaged, and subjected to quality control procedures intended to preserve viability throughout the anticipated shelf life [12]. Each manufacturing stage can affect cell viability, physiological status, and the technological performance of the final product, underscoring the need for carefully controlled processing conditions and continuous quality monitoring [12]. Microbiological quality testing, viability assessment, stability evaluation, and batch-to-batch reproducibility are therefore essential components of probiotic manufacturing rather than merely post-production verification steps [7]. Alongside accurate genomic identification and safety assessment, these manufacturing attributes provide the basis for the comprehensive characterization of probiotic strains intended for research and commercial applications [1,12].

Although numerous studies have investigated various biological and technological aspects of *L. reuteri*, these subjects are typically examined in isolation [1,8,9,12]. A significant knowledge gap exists regarding integrated evaluation frameworks that simultaneously link manufacturing quality, genomic identity, and preclinical safety for individual strains. To address this gap and establish a comprehensive baseline for a novel candidate, the present study aimed to perform an integrated, strain-specific characterization of the human-derived *L. reuteri* ATA-LTC-Lr050600. By combining controlled production and real-time stability monitoring with whole-genome sequencing, in vitro functional assays, and OECD-compliant in vivo toxicity studies, this research provides a unified dataset to evaluate the strain as a reproducible, technologically stable, and preclinically safe probiotic candidate.

## 2. Materials and Methods

### 2.1. Study Design

This study evaluated the production process, quality characteristics, and safety profile of the human-derived probiotic strain *L. reuteri* ATA-LTC-Lr050600. The assessment was based on validated manufacturing and analytical records, including fermentation and downstream processing, microbiological quality control, whole-genome sequencing (WGS), in vitro safety and functional analyses, and OECD-compliant acute and repeated-dose oral toxicity studies. Production was carried out under an ISO 22000-certified [16] food safety management system in controlled clean-room facilities compliant with ISO 14644-1 [17].

### 2.2. Strain Origin and Cell Banking

*L. reuteri* ATA-LTC-Lr050600 was isolated from a healthy human fecal sample collected in Balıkesir, Türkiye, and deposited in the ATA Culture Collection (catalog no. 159-RE). This specific strain was selected for the current study based on its human origin and promising preliminary screening results, which indicated robust growth kinetics and an absence of natural antibiotic resistance, making it an ideal candidate for industrial scale-up. Strain identity and traceability were confirmed through culture collection records and whole-genome sequencing. Master and working cell banks were maintained at −80 °C and under cryogenic storage conditions. Before fermentation, the strain was aseptically revived from the master cell bank, and its identity, purity, and viability were confirmed prior to seed culture preparation. Specifically, strain identity was definitively confirmed at the genomic level using Whole-Genome Sequencing (WGS) and Average Nucleotide Identity (ANI) analysis. Culture purity was strictly verified via Gram staining, microscopic examination, and the observation of uniform colony morphology on de Man, Rogosa and Sharpe (MRS) agar plates. Finally, cell viability was quantified using standard plate counting methods (expressed as CFU/mL) on MRS agar. Viable cell enumeration was performed using an automated colony counter (Interscience Scan 4000, Interscience, Saint-Nom-la-Bretèche, France) operated with its corresponding image analysis software (Scan^®^ software v8.0). The software settings were specifically optimized for lactobacilli colonies, and all automated outputs were subjected to manual visual verification by an experienced microbiologist to correct for overlapping colonies and ensure absolute counting accuracy.

### 2.3. Fermentation Medium and Seed-Culture Preparation

The production medium consisted of an animal-origin-free MRS-type formulation containing vegetable peptone, yeast extract, dextrose, plant-derived Tween 80, dipotassium hydrogen phosphate, manganese sulfate, magnesium sulfate, ammonium citrate, and sodium acetate. The medium was formulated according to the nutritional principles of de Man–Rogosa–Sharpe (MRS) medium for lactobacilli while replacing animal-derived ingredients with food-grade plant-based alternatives [18]. All raw materials complied with food-grade quality specifications and were accompanied by certificates confirming their non-animal origin.

Frozen stock cultures of *L. reuteri* ATA-LTC-Lr050600 were aseptically revived on selective culture medium and incubated under anaerobic conditions to obtain fresh pre-cultures. Following confirmation of culture purity and normal colony morphology, seed cultures were prepared until a viable cell density of at least 1 × 10^9^ CFU/mL was achieved. The production bioreactor was inoculated with 5% (*v*/*v*) seed culture, and fermentation was carried out under anaerobic conditions for 18–24 h using standard aseptic procedures. Throughout the fermentation process, microbiological contamination, culture integrity, and critical process parameters were routinely monitored to ensure batch consistency.

Samples collected at the end of fermentation were serially diluted in Maximum Recovery Diluent (MRD; Merck, Darmstadt, Germany). Decimal dilutions were prepared up to 10^−10^, and appropriate dilutions were spread onto selective agar plates using the Drigalski spread-plate technique. Plates were incubated at 37 °C for 72 h under anaerobic conditions. Plates containing 30–300 colonies were selected for enumeration using a calibrated colony counter, and viable cell counts were calculated by applying the corresponding dilution factor. Results were expressed as colony-forming units (CFU/mL) and recorded as part of the routine batch quality-control procedure.

### 2.4. Bioreactor Fermentation and In-Process Controls

Anaerobic seed cultures were inoculated at 5% (*v*/*v*) into a 5-L automated stirred-tank bioreactor [BioFlo 120, Eppendorf, Wesseling, Germany] operated in batch mode with a working volume of 4.0–4.5 L. The system was equipped with industry-standard monitoring instruments, including a sterilizable glass pH electrode [InPro 3100/325/PT1000, Mettler Toledo, Greifensee, Switzerland] and a temperature sensor. Culture media and all process-contact materials were sterilized by autoclaving at 121 °C for 10 min before use. Fermentation was performed in vegan de Man, Rogosa, and Sharpe (MRS) medium under aseptic anaerobic conditions for 18–24 h.

Critical fermentation parameters were continuously monitored and regulated to ensure batch consistency and product quality. The temperature was maintained at 37 ± 1 °C via a heating/cooling jacket. The initial pH was adjusted to 6.2 ± 0.2, and anaerobic conditions were maintained using standard inert gas sparging. During fermentation, the pH was continuously maintained within the operational range of 4.5–6.5 through the automated dosing of food-grade alkaline solutions. Agitation was maintained between 150 and 250 rpm to ensure homogenous mixing, and standard food-grade antifoam agents were applied dynamically as required to prevent excessive foaming. The entire fermentation process was conducted under controlled clean-room conditions using a closed bioreactor system.

At the completion of fermentation, samples were serially diluted in Maximum Recovery Diluent (MRD) up to $10^{-10}$. Appropriate dilutions were spread onto selective agar plates using the Drigalski spread-plate method and incubated at 37 °C for 18–24 h under anaerobic conditions. Enumeration of viable lactic acid bacteria was performed in accordance with ISO 15214. Plates containing 30–300 colonies were selected for enumeration, and viable cell counts were calculated using the corresponding dilution factor, expressed as colony-forming units per milliliter (CFU/mL).

Following fermentation, the biomass was recovered by centrifugation and subjected to downstream processing, including protective coating, freezing, and lyophilization. Cryoprotectant was applied separately to cultures intended for storage at −80 °C. All process observations, critical parameter records, viable cell counts, and batch-release results were documented within the routine manufacturing quality-control system.

### 2.5. Biomass Recovery, Washing, and Alginate Coating

Fermented biomass was recovered by mechanical separation/centrifugation and concentrated. The cell pellet was washed to separate the biomass from the liquid medium and reduce residual vegetable peptone, yeast extract, dextrose, Tween 80, phosphate, manganese, magnesium, ammonium citrate, and sodium acetate. The number and volume of washing cycles are controlled manufacturing parameters and were not disclosed in the public dossier.

The washed biomass was processed with plant-derived sodium alginate (E401; Merck/Sigma-Aldrich, Darmstadt, Germany, documentation in the dossier) as the protective coating material prior to freezing and lyophilization. The alginate specification included lead not exceeding 5 ppm, arsenic not exceeding 3 ppm, loss on drying below 15%, microbiological purity criteria, and declarations supporting non-animal origin and absence of BSE/TSE risk. Transfer between unit operations was performed using traceable containers and controlled material-status labeling.

### 2.6. Freezing, Lyophilization, and Dry Blending

Following biomass recovery, the coated probiotic cell concentrate was frozen at −80 °C before lyophilization. Freeze-drying was performed using a conventional three-stage process consisting of pre-freezing, primary drying under reduced pressure to facilitate ice sublimation, and secondary drying to remove residual moisture. This process was designed to preserve cell integrity and maintain probiotic viability during long-term storage.

Critical process variables, including shelf temperature, condenser temperature, vacuum pressure, and drying end-point, were monitored throughout the lyophilization cycle. Although the exact operating set points are proprietary, all parameters were maintained within validated manufacturing limits to ensure reproducible drying performance and low residual moisture in the final product. The lyophilization procedure followed established principles for the preservation and stabilization of probiotic microorganisms through controlled freezing and sublimation [19,20].

Following lyophilization, the dried biomass was blended with dry excipient materials under controlled low-moisture conditions to obtain a homogeneous probiotic powder. Dry blending was performed to ensure uniform distribution of the lyophilized cells throughout the bulk material while minimizing moisture exposure. After blending, the final product was evaluated for homogeneity and compliance with predefined moisture specifications before transfer to the packaging stage.

### 2.7. Bulk Packaging, Storage, and Stability Monitoring

The finished probiotic powder was packaged as a bulk raw material in moisture-protective primary packaging under controlled manufacturing conditions. Packaged products were stored at 25 ± 1 °C, protected from direct light and moisture, in accordance with the validated storage conditions established for the product [21].

Product stability was evaluated by real-time stability monitoring for approximately 15 months. At predefined intervals, viable cell counts were determined in accordance with ISO 15214, and the physical characteristics of the product, including appearance and moisture-related quality attributes, were assessed to verify compliance with the established product specifications. Stability was considered acceptable when all predefined microbiological and physical quality criteria remained within the specified acceptance limits throughout the monitoring period [22].

### 2.8. Viable Count and Microbiological Quality

Total viable probiotic bacteria were enumerated in accordance with ISO 15214 using the plate-count method for mesophilic lactic acid bacteria. Aerobic mesophilic microorganisms were determined according to ISO 4833-1 [23] yeasts and molds were enumerated according to ISO 21527-1 [24] or ISO 21527-2 [25], as appropriate for the product water activity. *Staphylococcus aureus* was examined according to ISO 6888-1 [26], and β-glucuronidase-positive Escherichia coli was determined according to ISO 16649-2 [27]. Product quality was further assessed by evaluating appearance, physical state, color, and odor in accordance with the established product specifications.

### 2.9. Whole-Genome Identity and Genomic Safety Screening

Whole-genome sequencing (WGS) was performed to confirm strain identity, assess genome assembly quality, and screen for antimicrobial resistance determinants and other genomic safety-related features. Genomic DNA was extracted from the pure culture of *Limosilactobacillus reuteri* ATA-LTC-Lr050600 using a semi-automated Kurabo Mini-80 system (KURABO, Osaka, Japan). DNA libraries were prepared using the Nextera XT DNA Library Preparation Kit and sequenced on an Illumina short-read sequencing platform (Illumina, Inc., San Diego, CA, USA).

Raw FASTQ files were used for downstream bioinformatic analysis. *De novo* genome assembly was performed using SPAdes v3.15 [28] with the “--isolate” parameter, and assembly quality was assessed using QUAST v5.2.0 [29]. Antimicrobial resistance genes were screened using the ResFinder database [30]. Functional genome annotation, including enzyme and metabolic pathway mapping, was performed using the GhostKOALA program against the KEGG database [31]. The assembled genome is accessible via the NCBI database (BioProject: PRJNA1092961, BioSample: SAMN40643421) under the RefSeq assembly accession GCF_037966725.1 (https://www.ncbi.nlm.nih.gov/datasets/genome/GCF_037966725.1/, accessed on 16 September 2026).

Standardized phenotypic MIC testing was not performed in the present study; consequently, the antimicrobial resistance assessment appears to rely on WGS-based screening of acquired resistance determinants, a limitation which necessitates cautious interpretation [32,33].

### 2.10. In Vitro Safety and Gastrointestinal Functional Assessments

Strain-specific in vitro studies were performed to evaluate cytotoxicity, hemolytic activity, gastrointestinal stress tolerance, and adhesion-related characteristics. Cytotoxicity was assessed according to ISO 10993-5 using CCD-18Co (ATCC) human colon fibroblast cells. While ISO 10993-5 commonly recommends standard reference lines such as L929 murine fibroblasts, the human colon fibroblast cell line CCD-18Co (ATCC CRL-1459) was specifically selected for this assay. Because *L. reuteri* ATA-LTC-Lr050600 is an orally administered probiotic intended to interact with the human gut mucosa, utilizing a human colon-derived cell line provides a highly physiologically relevant model for assessing target-tissue cytotoxicity. To ensure the validity and compliance of the assay on this specific cell line, standard positive (e.g., SDS or DMSO) and negative (culture medium) controls were strictly employed in accordance with ISO 10993-5 guidelines [34,35]. Cells were exposed to *L. reuteri* ATA-LTC-Lr050600 at approximately 2 × 10^6^ CFU/mL (100 µL per well) for 24 h, and cell viability was determined using the MTT assay. Dulbecco’s phosphate-buffered saline and 2.3% sodium dodecyl sulfate served as the negative and positive controls, respectively. All assays were performed in triplicate.

Adhesion-related capacity was evaluated by inoculating confluent CCD-18Co cell cultures with approximately 2 × 10^6^ CFU/mL bacterial suspension and incubating the cultures for 24 h at 37 °C under 5–15% CO_2_. Bacterial adhesion was examined microscopically, while post-incubation survival was assessed by viable-count enumeration following plating on Brain Heart Infusion agar.

Hemolytic activity was evaluated phenotypically on sheep blood agar. Ten parallel inoculations of *L. reuteri* ATA-LTC-Lr050600 were performed and incubated at 37 °C under anaerobic conditions for 48–72 h. *Staphylococcus aureus* ATCC 6538 was included as a positive control for β-hemolysis. After incubation, each inoculation site was examined visually for the presence or absence of a zone of erythrocyte lysis surrounding the bacterial growth. Complete absence of a visible hemolytic zone was classified as γ-hemolysis, whereas a clear zone of erythrocyte lysis would indicate β-hemolysis. The hemolytic phenotype was recorded for all ten parallel inoculations.

Tolerance to simulated gastrointestinal conditions was assessed by exposing bacterial suspensions (5 × 10^3^–5 × 10^6^ CFU/mL) to BHI medium adjusted to pH 1–5 with hydrochloric acid for 1–2 h and to BHI medium containing 0.1–0.5% (*w*/*v*) bile salts for 12 h at 37 °C under anaerobic conditions. Viable counts after exposure were used to determine survival under acid and bile stress.

### 2.11. Animal Studies

#### 2.11.1. Ethical Approval and Animal Husbandry

The animal study was conducted and reported in accordance with the ARRIVE 2.0 guidelines and OECD Guideline 407 for repeated-dose 28-day oral toxicity studies. Acute oral toxicity and repeated-dose oral toxicity studies were performed in accordance with OECD Test Guidelines 423 and 407, respectively. Both study protocols were approved by the İzmir Biomedicine and Genome Center Local Animal Experiments Ethics Committee (Protocol No. 2025-011; approval date: 10 April 2025).

Determination of group size adhered to OECD Guideline 407 and 423, which recommends five animals of each sex per experimental cohort. Specific pathogen-free Wistar albino rats (six to eight weeks of age; 180–220 g) were obtained from a certified breeding facility and underwent a seven-day acclimatization period prior to study initiation.

Animals were housed individually in ventilated stainless-steel cages under controlled environmental conditions (22 ± 3 °C, 50 ± 20% relative humidity, 12 h light/12 h dark cycle). Standard laboratory rodent chow and drinking water were provided ad libitum, and environmental enrichment was supplied throughout the study.

#### 2.11.2. Experimental Design

Acute oral toxicity was evaluated in Wistar albino rats (six females) according to OECD Test Guideline 423 using the limit-test procedure at a dose of 2000 mg/kg [36]. The repeated-dose study was conducted in Wistar albino rats according to OECD Test Guideline 407, with oral administration of 1000 mg/kg/day (approximately 1.2 × 10^11^ CFU/kg/day) for 28 consecutive days [37].

Animals were randomly assigned to negative control (NC) and *L. reuteri* ATA-LTC-Lr050600 treatment groups, each consisting of five males and five females. The probiotic preparation or vehicle (drinking water) was administered once daily by oral gavage for 28 consecutive days. Dose volume was adjusted weekly according to individual body weight measurements. At the end of the treatment period, animals were euthanized for toxicological evaluation. Additional recovery groups, each consisting of five female and five male rats, were maintained for a 14-day treatment-free recovery period to assess the reversibility or persistence of any treatment-related effects.

#### 2.11.3. Blood Collection, Hematological Analyses and Toxicological Evaluation

At the end of the 28-day treatment period and after the 14-day recovery period, animals were fasted overnight while water remained available ad libitum. Deep anesthesia was induced with inhaled isoflurane, and anesthetic depth was confirmed by the absence of the pedal withdrawal reflex in response to a firm toe pinch. While the animals remained under deep anesthesia, blood was collected by cardiac puncture. Euthanasia was completed by exsanguination followed by cervical dislocation before necropsy.

Blood intended for hematological analyses was collected into EDTA-treated tubes, while blood for serum biochemistry was collected into serum separator tubes. Hematological parameters, including white blood cell count (WBC), lymphocytes (LYM), monocytes (MON), granulocytes (GRA), red blood cell count (RBC), hemoglobin (HGB), hematocrit (HCT), and platelet count (PLT), were determined using an automated hematology analyzer (Boule Exigo H400, Boule Medical AB, Spånga, Sweden). Serum biochemical parameters evaluated included alanine aminotransferase (ALT), aspartate aminotransferase (AST), alkaline phosphatase (ALP), blood urea nitrogen (BUN), creatinine, total protein, albumin, glucose, total cholesterol, and triglycerides.

For histopathological evaluation, target organs (liver, kidneys, spleen, heart, and lungs) were rapidly excised, weighed, and immediately fixed in 10% neutral buffered formalin for 48 h. Following fixation, tissues were dehydrated through a graded ethanol series, cleared in xylene, and embedded in paraffin blocks. Tissue sections (4–5 µm thickness) were obtained using a rotary microtome (Leica RM2235 Rotary Microtome, Leica Biosystems, Nussloch, Germany), deparaffinized, and stained with Hematoxylin and Eosin (H&E) according to standard protocols. Histopathological evaluation was performed by a blinded veterinary pathologist under a light microscope (Olympus CX43 Biological Microscope, Olympus Corporation, Tokyo, Japan). Lesions were scored using a standardized, semi-quantitative 5-tier grading system *Shackelford* [38]: Grade 0 = normal/no lesion; Grade 1 = minimal (affecting <10% of the tissue); Grade 2 = mild (10–25%); Grade 3 = moderate (26–50%); and Grade 4 = severe (>50%).

### 2.12. Statistical Analysis

All statistical analyses were performed using [GraphPad Prism version 9.0 (GraphPad Software, Boston, MA, USA)]. Data are expressed as the mean ± standard deviation (SD). In all experiments, *n* represents the number of independent biological replicates, and the exact *n* value for each experiment is explicitly stated in the corresponding figure legends and tables. Prior to statistical comparisons, the normality of data distribution was assessed using the Shapiro–Wilk test, and the homogeneity of variances was evaluated using Levene’s test.

Based on these assessments, comparisons between two independent groups were performed using the unpaired Student’s *t*-test for normally distributed data, whereas the non-parametric Mann–Whitney U test was applied for non-normally distributed datasets. For comparisons involving more than two groups, a One-Way Analysis of Variance (ANOVA) was performed, followed by Tukey’s honestly significant difference (HSD) post hoc test to correct for multiple comparisons. All experimental data consisted of independent measurements; no paired or repeated-measures designs were employed. A *p*-value of <0.05 was considered statistically significant. The specific statistical test applied to each dataset is explicitly detailed in the respective figure legends.

## 3. Results

### 3.1. Fermentation Performance

*L. reuteri* ATA-LTC-Lr050600 was cultivated in vegan MRS medium in a 5 L automated bioreactor operated in batch mode as detailed in Table 1. The working volume was 4.0–4.5 L, the temperature was maintained at 37 ± 1 °C, and the initial pH was 6.2 ± 0.2. During fermentation, the pH remained between 4.5 and 6.5, while agitation was maintained at 150–250 rpm. The process was completed within 18–24 h and produced a final viable cell concentration of 1 × 10^11^ CFU/mL (11.0 log CFU/mL). No critical deviation affecting the evaluated batch was identified in the available production records.

After fermentation, the biomass was recovered by centrifugation and transferred to the downstream processing steps, including protective coating, freezing, and lyophilization. Cryoprotectant was used separately for cultures intended for storage at −80 °C. The final product was obtained as a homogeneous lyophilized probiotic powder suitable for bulk packaging.

Cryoprotectant supplementation was used specifically for cultures intended for storage at −80 °C and was not part of the lyophilization formulation.

### 3.2. Strain Identity, Viable Count, and Microbiological Quality

Whole-genome sequencing identified the analyzed strain as *L. reuteri* ATA-LTC-Lr050600, with no detectable microbial contamination in the sequencing dataset. The genome sequence was deposited in the NCBI BioSample database (BioSample accession SAMN40643421; BioProject PRJNA1092961). Taxonomic assignment was supported by the NCBI BioSample record, while antimicrobial resistance genes were evaluated using the ResFinder database. No acquired antimicrobial resistance determinants or evidence of contamination were identified in the analyzed genome. The complete WGS report and associated genomic analysis outputs are provided as Supplementary File S1.

The final lyophilized product contained 5.10 × 10^11^ CFU/g viable probiotic bacteria, as determined in accordance with ISO 15214. The corresponding fermentation and viable-count data are summarized in Table 1. Yeasts, molds, *Escherichia coli*, and coliform bacteria were not detected under the applied test conditions. The product also showed the expected appearance, physical state, color, and odor of a lyophilized probiotic powder. Together, these findings confirmed strain identity, high viable-cell concentration, and acceptable microbiological quality of the evaluated batch.

### 3.3. Stability and Carrier Quality

Real-time stability testing demonstrated that *L. reuteri* ATA-LTC-Lr050600 maintained a stable viable-cell count throughout 15 months of storage under the recommended conditions (25 ± 1 °C, protected from moisture and light). Viable counts remained between 9.6 and 9.7 log CFU/g, with only minor month-to-month fluctuations and no evidence of a marked decline during the study period (Figure 1). The slight downward trend observed over time remained within the predefined product acceptance criteria, supporting the stability of the lyophilized formulation during long-term storage.

Changes in viable cell counts (log CFU/g) of lyophilized *L. reuteri* ATA-LTC-Lr050600 during 15 months of real-time storage under the recommended storage conditions (25 ± 1 °C, protected from moisture and light). The dotted line represents the linear trend of viable cell counts over the storage period. Data are presented as the mean ± SD of five independent biological replicates (n = 5). Statistical significance among different time points was determined using One-Way ANOVA followed by Tukey’s post hoc test.

The sodium alginate coating material and chicory-derived inulin complied with the predefined quality specifications. The inulin contained 96.3% dry matter and 92.6% inulin, while lead and arsenic concentrations remained below the specified limits and *Salmonella* spp. and *Escherichia coli* were not detected. These findings confirmed the suitability of the excipients used in the final probiotic formulation.

### 3.4. In Vitro Safety and Functional Accessibility

No hemolytic activity was detected for *L. reuteri* ATA-LTC-Lr050600 in the phenotypic sheep blood-agar assay. All ten parallel inoculations showed no visible zone of erythrocyte lysis after incubation at 37 °C under anaerobic conditions for 48–72 h, corresponding to a γ-hemolytic phenotype under the applied test conditions. *Staphylococcus aureus* ATCC 6538, used as the positive control, exhibited the expected β-hemolytic reaction, confirming the validity of the assay. Cytotoxicity was evaluated according to ISO 10993-5 using CCD-18Co human colon fibroblast cells. Cell viability remained high following 24 h of exposure, with values of 99.1 ± 5.61% and 99.0 ± 3.09% for the two independent test preparations, comparable to the negative control and indicating no detectable cytotoxic effect (Figure 2A).

The strain also demonstrated good adhesion-related performance. Following incubation with CCD-18Co cells, viable bacterial counts were 1.76 ± 0.23 × 10^6^ CFU/mL after the first enumeration and 1.92 ± 0.09 × 10^6^ CFU/mL after the second enumeration, compared with an initial inoculum of 2.00 ± 0.00 × 10^6^ CFU/mL, indicating substantial retention of viable bacteria during the assay (Figure 2B).

To evaluate manufacturing consistency, the fermentation process was performed across three independent production batches. Final viable cell concentrations showed limited batch-to-batch variation, ranging from 10.9 to 11.1 log CFU/mL, while final pH values remained stable across batches. These findings indicate consistent end-point production performance across the independent fermentation runs. Importantly, these fermentation batches were used to assess manufacturing reproducibility and were not the biological replicates represented in Figure 3, which presents the results of separate in vitro simulated gastrointestinal tolerance experiments. Continuous bacterial growth curves, such as time-resolved optical density or serial CFU measurements during fermentation, were not dynamically monitored. Instead, process control and batch reproducibility were assessed through real-time monitoring of critical physicochemical parameters, including pH, temperature, and agitation, together with end-point viable cell enumeration and post-fermentation quality testing. Because time-resolved substrate-consumption data and intermediate mass-balance measurements were not recorded, dynamic biomass productivity and intermediate recovery yields could not be determined.

Tolerance to simulated gastrointestinal conditions was evaluated using independent biological replicates, and the variability among replicates is presented as standard deviation in Figure 3. Under acidic conditions, mean survival increased from approximately 72% at pH 1.5–2.0 to 92% at pH 5.0 (Figure 3A). In the presence of bile salts, mean survival remained relatively high across the tested concentrations, ranging from approximately 90% to 94% (Figure 3B). The inclusion of error bars provides an estimate of experimental variability across the independent biological replicates.

### 3.5. Acute and Repeated-Dose Toxicity

Acute oral toxicity testing performed according to OECD Test Guideline 423 showed no mortality or treatment-related signs of severe toxicity following administration of the limit dose of 2000 mg/kg.

In the 28-day repeated-dose oral toxicity study conducted according to OECD Test Guideline 407, daily oral administration of 1000 mg/kg/day produced no treatment-related mortality, morbidity, or adverse clinical findings. Body weight, body weight gain, and food consumption remained comparable to those of the negative control group throughout the treatment and recovery periods, indicating no treatment-related effects on general growth or nutritional status (Figure 4).

Hematological evaluation included WBC, RBC, HGB, HCT, PLT, and coagulation time. WBC, PLT, and coagulation time remained comparable between the treatment and control groups in both sexes (Figure 5). Statistically significant differences were observed in selected erythrocyte-related parameters, specifically RBC, HGB, and HCT, in male animals. These differences were evaluated together with the overall toxicological findings, including clinical observations, body weight, food consumption, organ weights, gross pathological examination, and histopathology. No corresponding treatment-related abnormalities were identified in these complementary endpoints. Therefore, the isolated statistical differences were not considered sufficient evidence of a treatment-related hematological or hemolytic effect under the conditions of the study.

### 3.6. Overall Safety Assessment

The combined findings from strain identification, microbiological quality testing, whole-genome sequencing, in vitro characterization, real-time stability assessment, and in vivo toxicity studies consistently supported the safety profile of *L. reuteri* ATA-LTC-Lr050600. The strain maintained high viability throughout the 15-month storage period (Figure 1), showed no detectable cytotoxicity or hemolytic activity, tolerated simulated gastrointestinal conditions, and retained adhesion-related capacity in vitro (Figure 2 and Figure 3). In vivo evaluation demonstrated no treatment-related adverse effects following acute (OECD 423) or repeated-dose (OECD 407) oral administration, establishing a NOAEL of 1000 mg/kg/day under the conditions of the study (Figure 4 and Figure 5). Whole-genome sequencing confirmed strain identity and did not detect acquired antimicrobial resistance genes in the analyzed genome. Together, these findings support the microbiological quality and preclinical safety of the evaluated probiotic strain under the tested production and experimental conditions.

## 4. Discussion

This study assessed a single *L. reuteri* isolate for manufacturing reproducibility, genomic identity and preclinical safety within one experimental framework. Production was consistent across three independent runs, the genome matched the species type strain at 99.99% ANI with no acquired resistance determinants, and 28 days of oral dosing in rats produced no treatment-related effects. The sections below consider how far each result extends.

The assembly returned 99.99% ANI against the *L. reuteri* type strain DSM 20016, with 97.21% coverage, so there is no ambiguity about the species. Genome size (~2.0 Mb) and GC content (39.0%) sit within the range described for *L. reuteri* [6,7] and CheckM gave 98.76% completeness with 0.65% contamination—good enough for the screening we then applied, though it is a scaffold-level draft and we would not use it for comparative genomics without closing it first. No acquired resistance determinants were found. That result carries more weight in this species than it might elsewhere, because there is a well-documented precedent. *L. reuteri* ATCC 55730 had been in commercial use for some time before it was shown to carry two plasmids encoding *tet*(W) and *lnu*(A); the strain now sold as DSM 17938 is the plasmid-cured derivative made specifically to get rid of them [39]. That episode is largely why genomic screening for transferable determinants is now expected of any new candidate before it goes anywhere near a product [32,33]. ATA-LTC-Lr050600 was screened on that basis from the start. But the screen has a boundary that should be stated plainly. ResFinder finds acquired genes; it does not measure susceptibility. EFSA guidance asks for phenotypic MIC determination against a defined antibiotic panel before a strain can be called characterised, and we did not do this. What we can say is that no known transferable determinant is present. What we cannot say is that the strain is susceptible. Broth microdilution against the standard panel is the obvious next step and we regard it as unfinished business rather than as outside the scope of the work.

Survival was 72% after exposure at pH 1.5 and pH 2.0, rose to 82% at pH 3.0, and reached 92% at pH 5.0. Across bile salt concentrations of 0.1–0.5% it stayed between 90% and 94%, with no concentration-dependent decline over that range. Of the two, the acid figure is the more informative. Bile tolerance at these concentrations is common among intestinal lactobacilli and does not discriminate much between strains, whereas survival below pH 2 varies a good deal [5,9]. Comparing our numbers directly with published values for other *L. reuteri* strains is harder than it looks, because exposure time, inoculum density and matrix all differ between studies—a strain held for one hour at pH 2 in buffer and one held for three hours in simulated gastric fluid with pepsin are not being asked the same question. Recovery of DSM 17938 and ATCC 55730 from the human gastrointestinal tract after oral administration has been demonstrated [40,41], and our in vitro figures are at least consistent with that, but those were in vivo recovery studies and the comparison is weaker than the numbers make it appear. We would read our results as showing that the strain is not obviously acid-labile. We would not read more into them than that.

After 24 h of co-incubation with CCD-18Co cells, viable counts were 1.76 ± 0.23 × 10^6^ and 1.92 ± 0.09 × 10^6^ CFU/mL against an inoculum of 2.00 × 10^6^ CFU/mL. Attachment in this species is generally attributed to mucus-binding proteins and sortase-anchored surface adhesins [42,43]. We have not shown that the corresponding genes are expressed in our strain, or that they mediate what we observed, and we do not want to imply otherwise. The model is a real limitation and we would rather raise it ourselves than have it raised for us. CCD-18Co is a colonic fibroblast line. Adhesion work in this field is usually done on Caco-2 or HT-29 monolayers, which form polarised epithelium and, in the mucus-secreting HT-29 MTX variant, present the mucin layer that these adhesins are thought to bind. Fibroblasts do neither. The assay therefore shows that the strain attaches to a human colon-derived cell surface under the conditions used; it says nothing reliable about adhesion to intestinal mucosa. Repeating it on a mucus-producing epithelial model is the correct next experiment.

Three independent runs gave final counts of 10.9–11.1 log CFU/mL, and the lyophilised material contained 5.10 × 10^11^ CFU/g. The narrow spread across runs is the point worth making here. Batch-to-batch variability is a recognised weakness in probiotic manufacture and is rarely reported at all [12,15], so we have reported it even though three runs is a small number. We did not follow growth kinetics during fermentation and did not record time-resolved substrate consumption, which means biomass productivity and intermediate yields cannot be derived from our data; process control rested on pH, temperature and agitation monitoring with end-point enumeration. One aspect of the formulation deserves a comment. The carrier used in dry blending is chicory inulin, which is a fermentable prebiotic rather than an inert bulking agent, so the finished powder is in effect a synbiotic composition. Combinations of this kind have been studied for DSM 17938, where prebiotic co-administration was evaluated for its effect on persistence and metabolic activity in the human gut [44]. We selected inulin for its technological function and we did not test whether it contributes anything of that sort here. We mention it because it would have to be addressed if the product were developed further, not because we are claiming a synbiotic effect. Counts in the packaged product held between 9.6 and 9.7 log CFU/g over 15 months at 25 °C. Loss of viability during ambient storage is the usual failure mode for freeze-dried lactic acid bacteria and is driven mainly by residual moisture and oxidative damage to membrane lipids [19,20]. Under half a log over fifteen months at room temperature is a reasonable result for an alginate-protected, low-moisture powder. It is also a result obtained in a controlled facility, which is not the same as distribution conditions, and we have not run accelerated stability or temperature-excursion testing.

The OECD-compliant acute and repeated-dose oral toxicity studies provided complementary in vivo evidence for the safety characterization of *L. reuteri* ATA-LTC-Lr050600 under the experimental conditions employed. No treatment-related mortality or adverse clinical observations were identified during the evaluation period, and the investigated strain produced no toxicologically relevant effects on body weight, food consumption, hematological parameters, organ weights, gross pathological examination, or histopathological assessment. The no-observed-adverse-effect level established under the repeated-dose study conditions further supports the absence of treatment-related toxicity at the highest administered dose evaluated. Isolated differences observed between study groups were not accompanied by corroborating pathological or clinical findings and were therefore not considered indicative of treatment-related adverse effects under the conditions and duration of the study. These observations support the absence of treatment-related toxicological findings under the tested conditions and complement, rather than replace, the genomic and in vitro evidence generated within the integrated strain-specific safety assessment.

These results sit alongside the clinical record for the species rather than substituting for it. DSM 17938 has been through a randomised masked safety and tolerability trial in healthy adults [45]; no comparable data exist for ATA-LTC-Lr050600. A 28-day rodent study covers subacute oral toxicity and nothing else—not chronic exposure, not reproductive or developmental endpoints, and not administration to compromised hosts.

The limitations are worth listing together. One strain was studied, so none of this generalises to *L. reuteri* as a species—which is precisely the argument for strain-level characterisation in the first place [1,2]. The in vitro models are simplified, and in the case of the adhesion assay they are not the ones normally used. Antimicrobial susceptibility was assessed genotypically only. Three fermentation runs is a small number on which to base a claim about reproducibility. No efficacy endpoint was measured, in animals or in humans, and no human data were generated at all. Head-to-head comparison with DSM 17938 under identical conditions, phenotypic MIC determination, and adhesion testing on an epithelial model would each add more to the characterisation than another descriptive dataset would.

## 5. Conclusions

This study characterised *L. reuteri* ATA-LTC-Lr050600 across production, genomic identity, in vitro behaviour, storage stability and rodent oral toxicity. Three fermentation runs produced material of consistent viable count, and the lyophilised product retained viability within specification for 15 months at 25 °C. Whole-genome sequencing confirmed the species assignment at 99.99% ANI to the type strain and detected no acquired antimicrobial resistance determinants; this is a genomic prediction, and phenotypic susceptibility testing has not been performed. In vitro, the strain tolerated simulated gastric and bile conditions, attached to a human colon fibroblast line, and showed neither haemolysis nor cytotoxicity. In rats, acute dosing at 2000 mg/kg and 28 days of dosing at 1000 mg/kg/day produced no treatment-related adverse effects, giving a NOAEL of 1000 mg/kg/day at the highest dose tested. No efficacy endpoint was measured, in animals or in humans, and none of these results should be read as evidence of a health benefit. Establishing that would require controlled human studies, which have not been conducted for this strain. Phenotypic antibiotic susceptibility testing, adhesion assays on an intestinal epithelial model, longer-term toxicity data and direct comparison with established reference strains are the steps needed before the strain can be regarded as fully characterised.

## Figures and Tables

**Figure 1:**
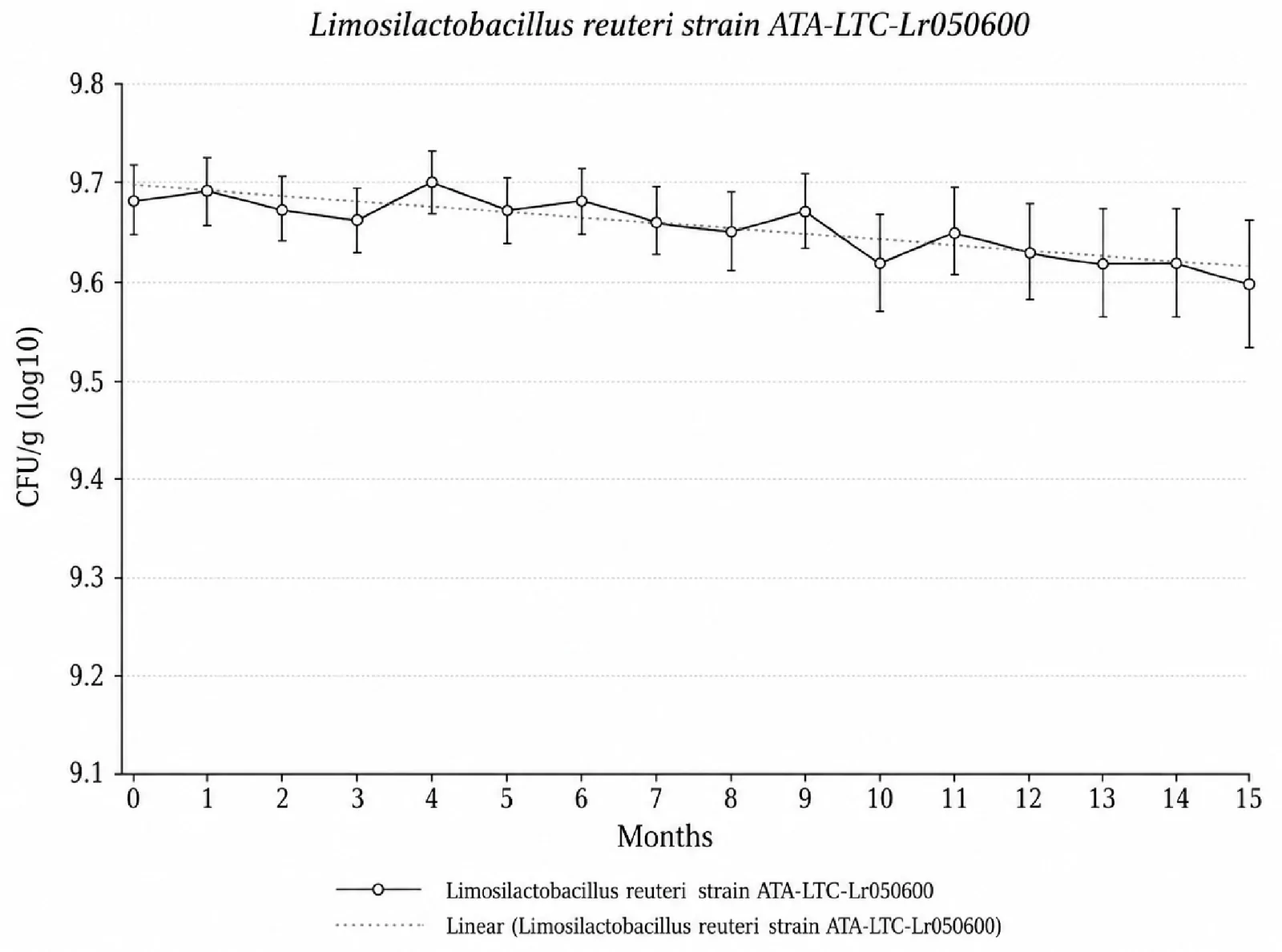
Real-time stability of *L. reuteri* ATA-LTC-Lr050600 during 15 months of storage at 25 ± 1 °C.

**Figure 2:**
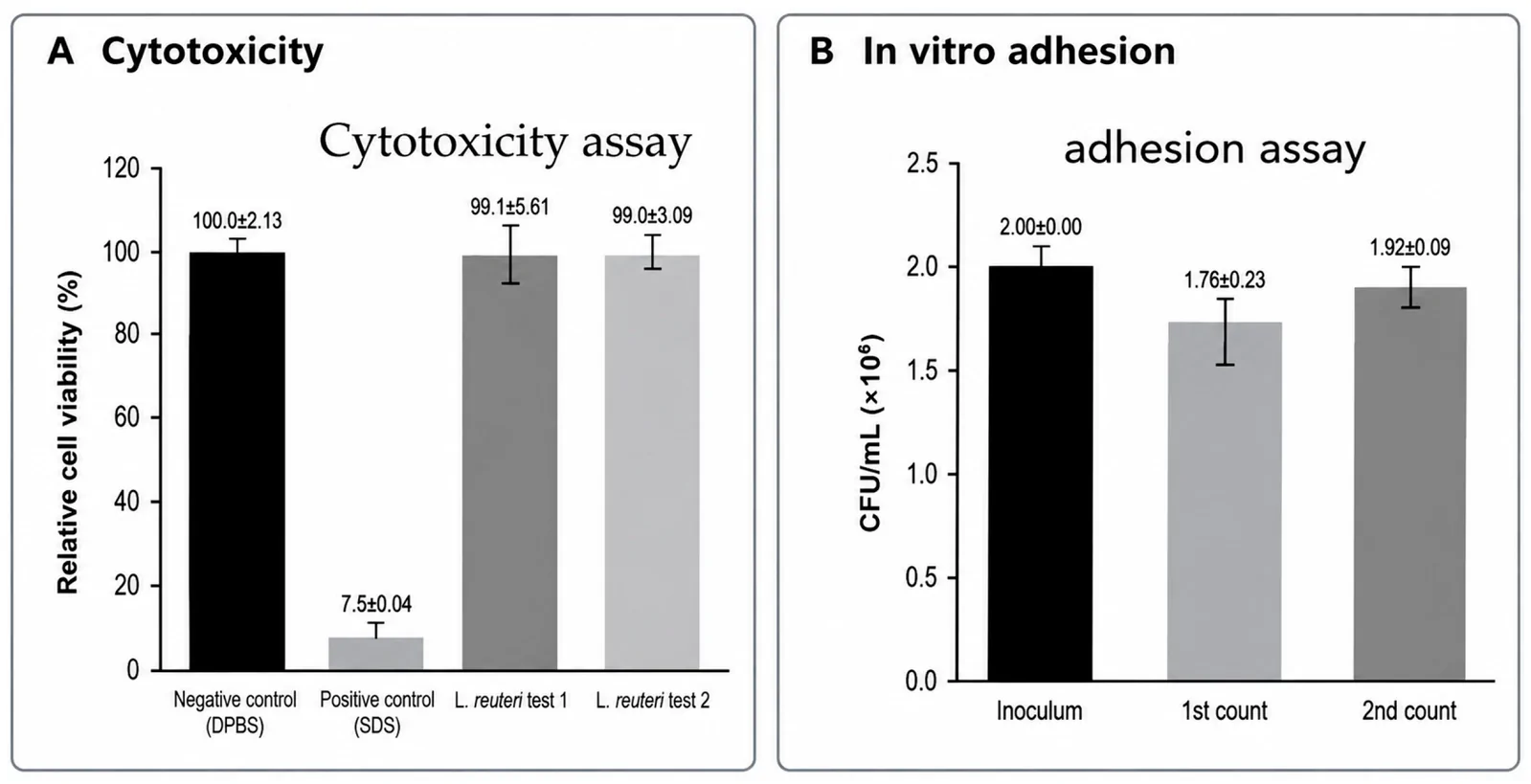
In vitro cytotoxicity and adhesion of *L. reuteri* ATA-LTC-Lr050600. (**A**) Cytotoxicity assessment of *L. reuteri* ATA-LTC-Lr050600 in CCD-18Co human colon fibroblast cells according to ISO 10993-5 guidelines. DPBS and SDS were used as the negative and positive controls, respectively. (**B**) In vitro adhesion assay showing viable bacterial counts before inoculation and after incubation with CCD-18Co cells. Data are presented as the mean ± SD of three independent biological replicates (n = 5). Statistical significance was determined using One-Way ANOVA followed by Tukey’s post hoc test for cytotoxicity (Panel **A**), and the unpaired Student’s *t*-test for adhesion comparisons (Panel **B**).

**Figure 3:**
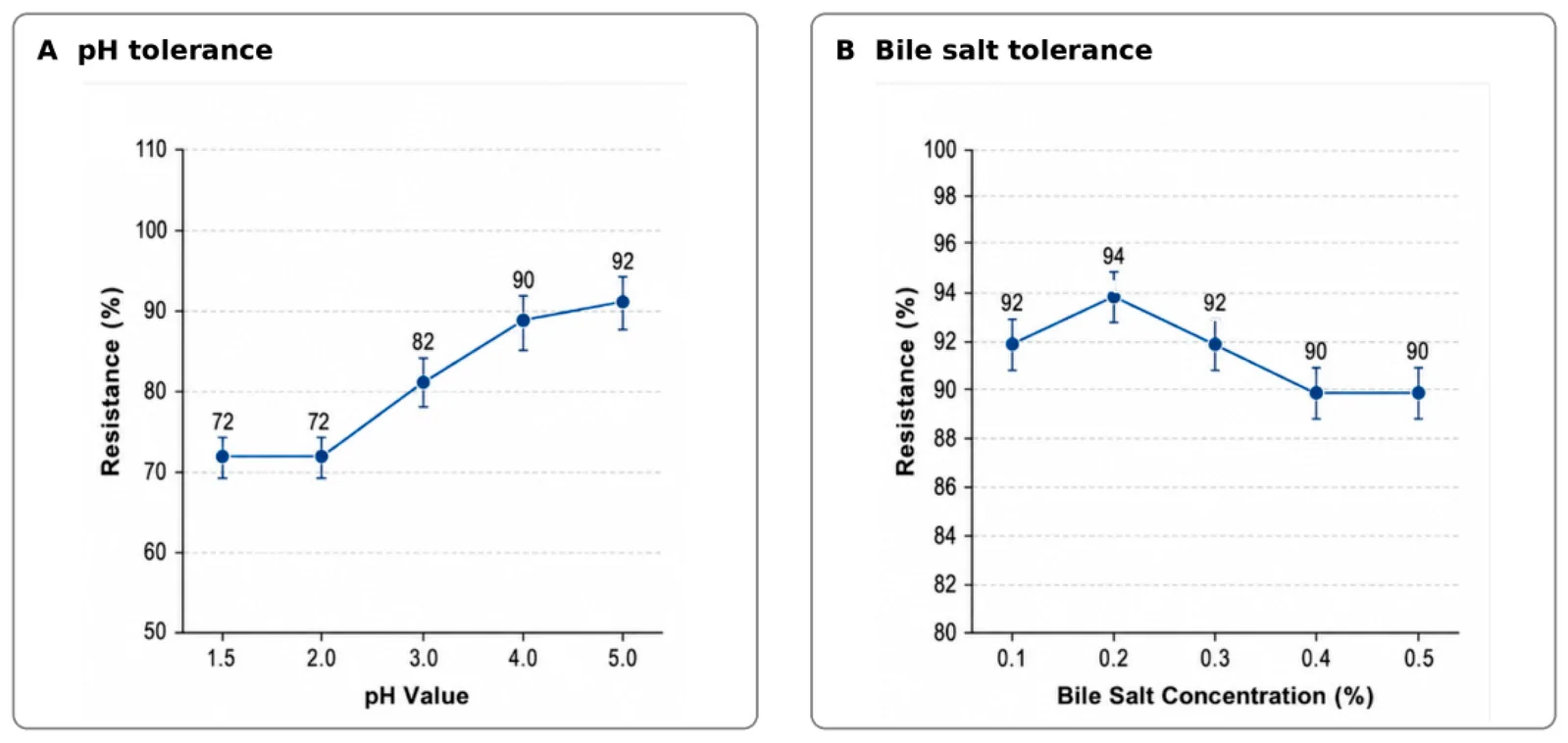
Survival of *Limosilactobacillus reuteri* ATA-LTC-Lr050600 under simulated gastrointestinal conditions. Survival of *L. reuteri* ATA-LTC-Lr050600 under simulated gastrointestinal conditions. (**A**) Survival of *L. reuteri* ATA-LTC-Lr050600 following exposure to different pH values. (**B**) Survival following exposure to increasing concentrations of bile salts (0.1–0.5%). Results are expressed as survival percentages relative to the initial viable count and are presented as mean ± SD of three independent biological replicates (n = 3). Error bars represent the standard deviation. Statistical significance among pH values and bile salt concentrations was determined using one-way ANOVA followed by Tukey’s post hoc test.

**Figure 4:**
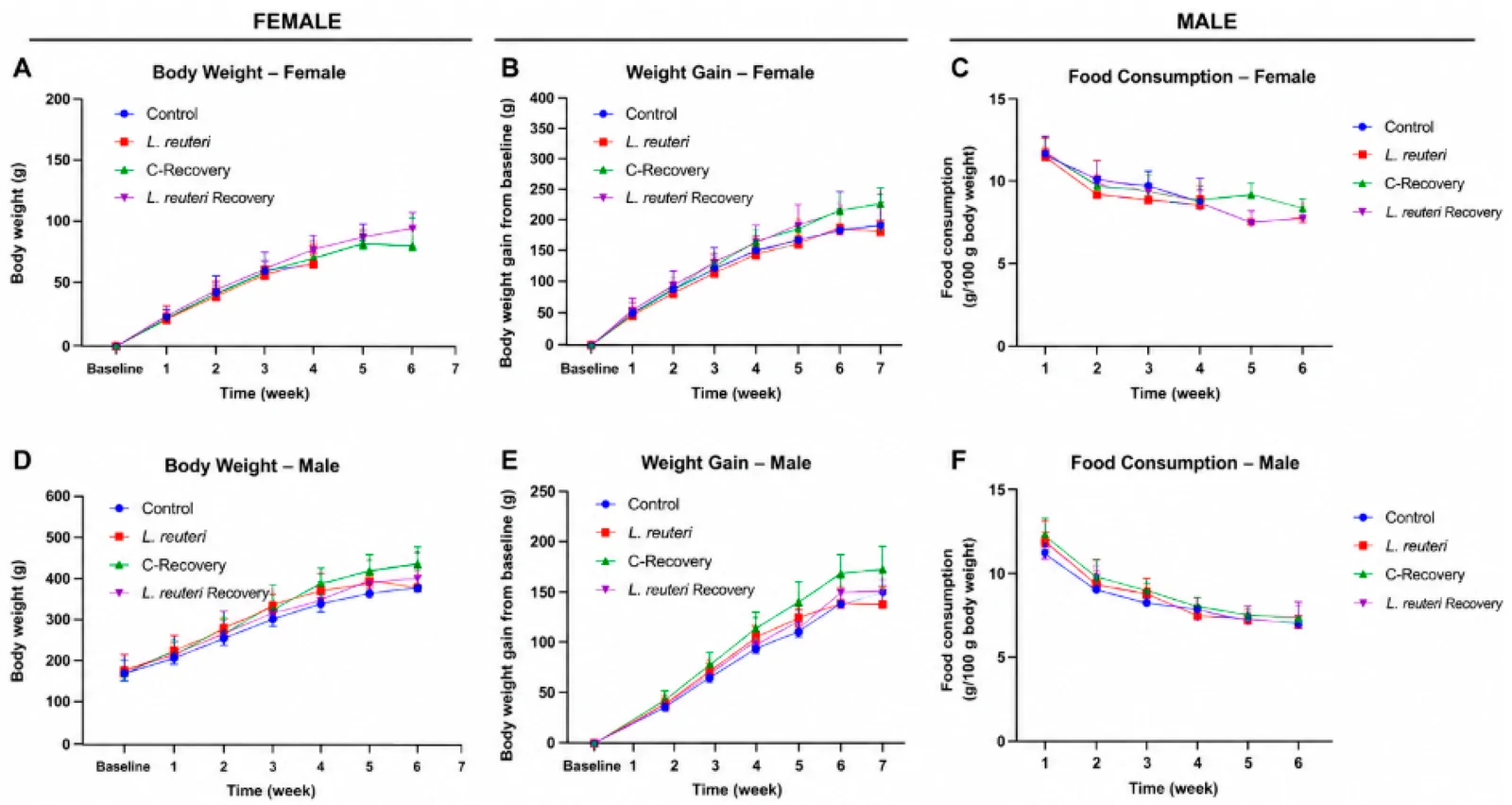
Effects of repeated oral administration of *Limosilactobacillus reuteri* ATA-LTC-Lr050600 on body weight, body weight gain, and food consumption in the OECD Test Guideline 407 study. Female and male body weight (**A**,**D**), body weight gain (**B**,**E**), and food consumption normalized to body weight (**C**,**F**) during the 28-day treatment period and recovery phase. NC, negative control; TM, test material. Recovery denotes animals evaluated after the treatment-free recovery period. Data are presented as mean ± SD (n = 5 animals per sex per group). Statistical significance was determined using the unpaired Student’s *t*-test for pairwise comparisons and one-way ANOVA for multi-group comparisons (Figure 4).

**Figure 5:**
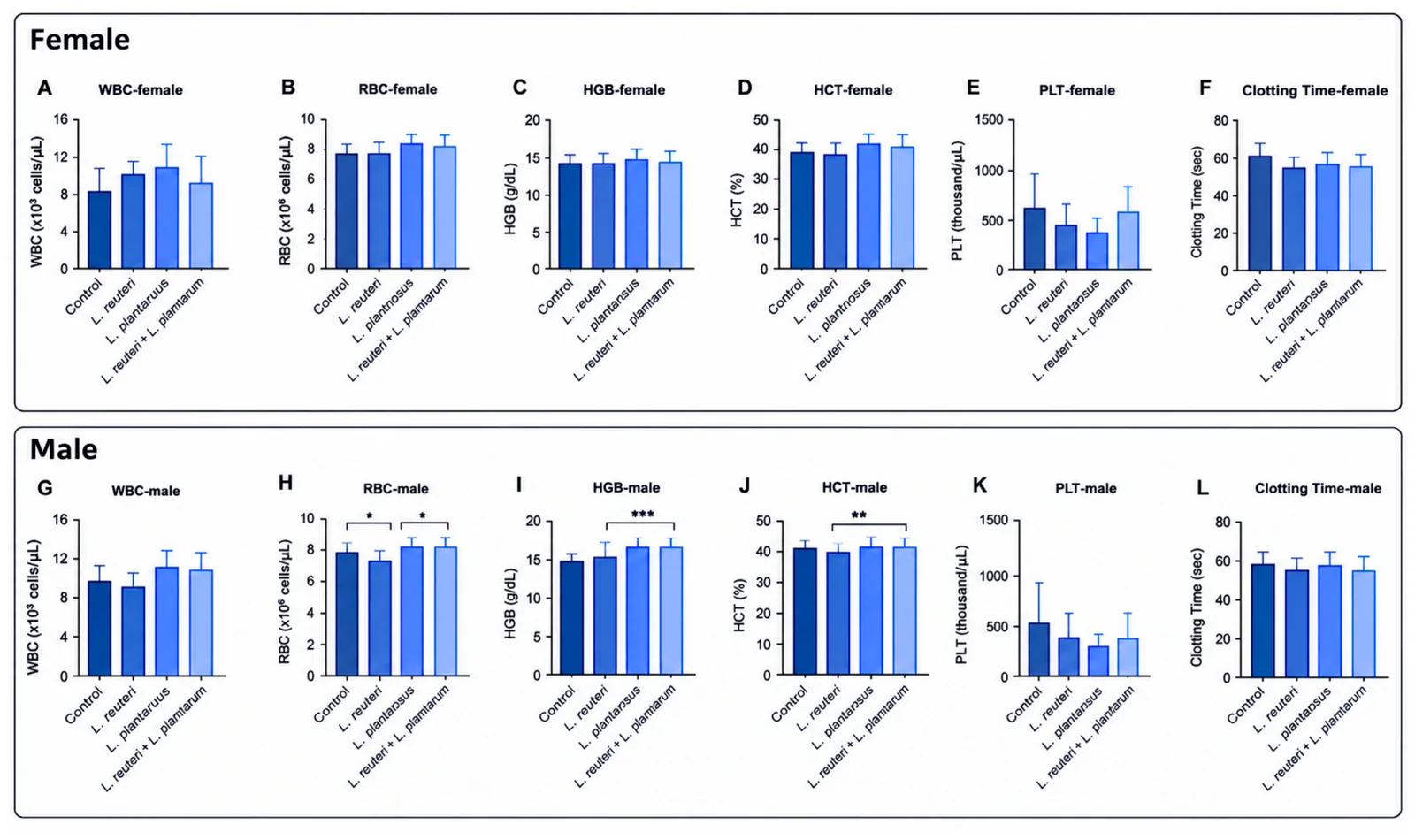
Hematological findings following repeated oral administration of *L. reuteri* ATA-LTC-Lr050600 in the OECD Test Guideline 407 study. Hematological findings following repeated oral administration of *L. reuteri* ATA-LTC-Lr050600 in the OECD Test Guideline 407 study. Female (**A**–**F**) and male (**G**–**L**) hematological parameters, including white blood cell count (WBC), red blood cell count (RBC), hemoglobin (HGB), hematocrit (HCT), platelet count (PLT), and coagulation time, determined at the end of the study. NC, negative control; TM, test material. Recovery denotes animals evaluated after the treatment-free recovery period. Data are presented as the mean ± SD (n = 5 animals per sex per group). Asterisks indicate statistically significant differences between the indicated groups (* *p* < 0.05, ** *p* < 0.01, *** *p* < 0.001). Statistical significance was determined using the unpaired Student’s *t*-test or One-Way ANOVA followed by Tukey’s post hoc test, as appropriate.

**Table 1:** Fermentation performance and downstream processing parameters for *L. reuteri* ATA-LTC-Lr050600.

Category	Parameter	Value
Operational Parameters	Fermentation temperature	37 °C
	pH control range	4.5–6.5
	Fermentation time	18–24 h
	Atmosphere	Anaerobic (Inert gas sparging)
	Base titrant	Food-grade alkaline solution
End-point Yields	Final viable cell concentration	10.9–11.1 log CFU/mL
	Post-fermentation viability	>95%
	Biomass recovery	High yield

## Data Availability

The data summarized in this manuscript are contained in the *L. reuteri* ATA-LTC-Lr050600 safety dossier and its associated validated reports. Raw WGS files, the NCBI BioSample accession, proprietary fermentation set points, and replicate-level analytical data are available from the corresponding author subject to legitimate commercial-confidentiality restrictions and journal review requirements. Genome data are openly available at NCBI under BioProject PRJNA1092961 (https://www.ncbi.nlm.nih.gov/bioproject/PRJNA1092961/, accessed on 16 September 2026), BioSample SAMN40643421 (https://www.ncbi.nlm.nih.gov/biosample/SAMN40643421/, accessed on 16 September 2026), WGS accession JBBMOQ000000000, and assembly accessions GCA_037966725.1 and GCF_037966725.1. Assembly statistics, annotation output, and resistance screening results are provided in Supplementary File S1. Replicate-level analytical records for the production, stability, and toxicity work are available from the corresponding author upon request.

## Supplementary Materials

The following supporting information can be downloaded at: https://www.mdpi.com/article/10.3390/microorganisms14102143/s1.

## Abbreviations

The following abbreviations are used in this manuscript:

AI	artificial intelligence
ARRIVE	Animal Research: Reporting of In Vivo Experiments
ATCC	American Type Culture Collection
BHI	Brain Heart Infusion
BSE	bovine spongiform encephalopathy
CFU	colony-forming unit
DNA	deoxyribonucleic acid
DPBS	Dulbecco’s phosphate-buffered saline
GRA	granulocytes
HCT	hematocrit
HGB	hemoglobin
ISO	International Organization for Standardization
LYM	lymphocytes
MIC	minimum inhibitory concentration
MON	monocytes
MRD	Maximum Recovery Diluent
MRS	de Man–Rogosa–Sharpe
MTT	3-(4,5-dimethylthiazol-2-yl)-2,5-diphenyltetrazolium bromide
NC	negative control
NCBI	National Center for Biotechnology Information
NOAEL	no-observed-adverse-effect level
OECD	Organisation for Economic Co-operation and Development
PLT	platelet count
RBC	red blood cell count
SD	standard deviation
SDS	sodium dodecyl sulfate
TM	test material
TSE	transmissible spongiform encephalopathy
WBC	white blood cell count
WGS	whole-genome sequencing
