# Supplementary material for: Integrated Production, Genomic Safety, Quality, and Stability Characterization of a Human-Derived Limosilactobacillus reuteri ATA-LTC-Lr050600 Strain for Probiotic Applications
Source: Microorganisms. 2026 Sep 23;14(10):2143. doi: 10.3390/microorganisms14102143 (PMC13707713; doi:10.3390/microorganisms14102143)
Supplement: Supplementary file 1 [file microorganisms-14-02143-s001.zip › microorganisms-4498335-supplementary.pdf]

ARRIVE 2.0: AUTHOR CHECKLIST

Compliance Table for Study GLP-2025-18

Category	material / Recommendation	Reporting Status / Description
**The ARRIVE Essential 10**	1. Study design	a. Specified in the text (Main Group and Rebound Groups; comparative design with a negative control group).<br>b. Individual rats (experimental unit) housed in conventional cages.
	2. Sample size	a. 10 female and 10 male rats per group (total number of animals specified in the text).<br>b. Determined in accordance with OECD Test Guideline No. 407 and the limit test approach.
	3. Inclusion and exclusion criteria	a. Criterion that live weight must be within ±20% of the average live weight determined for the respective sex.<br>b. No animals or data were excluded from the analysis within the scope of the study.<br>c. n = 10 for each group (females and males separately).
	4. Randomisation	a. Randomization and group allocation were performed.<br>b. They were housed in conventional cages under equivalent conditions.
	5. Blinding	Blinding was not implemented in this study due to the probiotic nature of the test substance and the limit test approach.
	6. Outcome measures	a. 28-day repeated-dose oral toxicity parameters: mortality-morbidity, clinical observations, and hematological/biochemical evaluations.<br>b. Systemic toxicity parameters mandated by the OECD 407 guideline.
	7. Statistical methods	a. Relevant statistical analysis methods and the software used.<br>b. Conformity of the data to normality assumptions.

	8. Experimental animals	a. Rat; species/status information, sex (female/male), age and weight limits.<br>b. Produced at the Izmir Biomedicine and Genome Center (IBG); conventional health status.
	9. Experimental procedures	a, b, c, d. Administration via oral gavage for 28 days in accordance with OECD Guideline 407; 5-day acclimatization period in room 4068 at the İBG, and the associated justifications.
	10. Results	a, b. Descriptive statistics and measures of central tendency and variability presented in individual tables and summary report data.
**The Recommended Set**	11. Abstract	The objectives, type, nature, fundamental methods, findings, and conclusions of the study are presented in the abstract.
	12. Background	a. Scientific background regarding the 28-day repeated-dose oral toxicity assessment of the probiotic product.<br>b. Relevance of the rodent model to human biology and toxicity testing.
	13. Objectives	Evaluation of the potential systemic toxic effects of the probiotic product.
	14. Ethical statement	Approval obtained from the IBG Local Ethics Committee for Animal Experiments, dated 10.04.2025 and bearing protocol number 2025-011.
	15. Housing and husbandry	Room 4068, conventional cages, IBG housing conditions.
	16. Animal care and monitoring	a. The 3R principles (Replacement, Reduction, Refinement) have been observed.<br>b, c. Observed clinical findings and monitoring frequency.
	17. Interpretation / scientific implications	a. Interpretation of the results in accordance with the literature and the OECD 407 guideline.<br>b. Study limitations (e.g., exclusion of optional parameters due to the limit test approach).

	18. Generalisability / translation	The generalizability of the findings to human biology and other species.
	19. Protocol registration	Information on the study protocol conducted under GLP conditions.
	20. Data access	Raw data tables (Annex-1).
	21. Declaration of interests	Conflict of interest and funding agency information.
